# Course of Cognitive Functioning in Institutionalized Persons With Moderate to Severe Dementia: Evidence From the Severe Impairment Battery Short Version

**DOI:** 10.1017/S1355617718000991

**Published:** 2018-11-20

**Authors:** Evelien T. Wolf, Wouter D. Weeda, Roland B. Wetzels, Jos F. M. de Jonghe, Raymond C. T. M. Koopmans

**Affiliations:** 1 Section Clinical Neuropsychology, Vrije Universiteit Amsterdam, Amsterdam, The Netherlands; 2 Department of Psychology, Leiden University, Leiden, the Netherlands; 3 Department of Primary and Community Care, Radboud University Medical Center, Nijmegen, The Netherlands; 4 Radboud Alzheimer Center, Nijmegen, The Netherlands; 5 Zzg, Elderly Care Organization, Nijmegen, The Netherlands; 6 Geriatric Medicine, North West Hospital Group, Alkmaar, The Netherlands; 7 Joachim en Anna, Centre for Specialized Geriatric Care, Nijmegen, The Netherlands

**Keywords:** Cognition, Cognitive decline, Dementia, Dementia stages, Institutionalization, Longitudinal studies, Neuropsychological tests

## Abstract

**Objectives:** To adequately monitor the course of cognitive functioning in persons with moderate to severe dementia, relevant cognitive tests for the advanced dementia stages are needed. We examined the ability of a test developed for the advanced dementia stages, the Severe Impairment Battery Short version (SIB-S), to measure cognitive change over time. Second, we examined type of memory impairment measured with the SIB-S in different dementia stages. **Methods:** Participants were institutionalized persons with moderate to severe dementia (*N* = 217). The SIB-S was administered at 6-month intervals during a 2-year period. Dementia severity at baseline was classified according to Global Deterioration Scale criteria. We used mixed models to evaluate the course of SIB-S total and domain scores, and whether dementia stage at baseline affected these courses. **Results:** SIB-S total scores declined significantly over time, and the course of decline differed significantly between dementia stages at baseline. Persons with moderately severe dementia declined faster in mean SIB-S total scores than persons with moderate or severe dementia. Between persons with moderate and moderately severe dementia, there was only a difference in the rate of decline of semantic items, but not episodic and non-semantic items. **Conclusions:** Although modest floor and slight ceiling effects were noted in severe and milder cases, respectively, the SIB-S proved to be one of few available adequate measures of cognitive change in institutionalized persons with moderate to severe dementia. (*JINS*, 2019, *25*, 204–214)

## INTRODUCTION

Dementia due to neurodegenerative disease is primarily characterized by a progressive deterioration of cognitive functioning (Grand, Caspar, & Macdonald, 2011). This deterioration eventually leads to increased dependency on others, and it is a strong indicator of institutionalization (Gaugler, Yu, Krichbaum, & Wyman, 2009). Consequently, the vast majority of persons with dementia reside in institutions 8 years after the initial dementia diagnosis (Luppa, Luck, Brähler, König, & Riedel-Heller, 2008). Institutionalized persons with dementia have on average more severe dementia than community dwelling persons with dementia (Boersma, Eefsting, Van Den Brink, & Van Tilburg, 1997), and relevant cognitive tests may be needed to monitor the course of cognitive functioning in more advanced dementia stages. In this way, pharmacological and non-pharmacological interventions targeting cognition, which are increasingly implemented in institutionalized persons with dementia, can be adequately evaluated.

One of the major problems with measuring the course of cognitive functioning in persons with dementia is floor and ceiling effects of cognitive tests. Although the Mini-Mental State Examination (MMSE; Folstein, Folstein, & McHugh, 1975) has often been used to monitor the course of cognitive functioning in persons with mild to moderate dementia (Galasko, Gould, Abramson, & Salmon, 2000; Thomas, Albert, Petersen, & Aisen, 2016), it shows a floor effect in more advanced dementia (Galasko et al., 2000). Several tests for advanced dementia have been developed. Examples of commonly used tests are the Severe Cognitive Impairment Profile (SCIP; Peavy et al., 1996), Severe Mini-Mental State Examination (SMMSE; Harrell, Marson, Chatterjee, & Parrish, 2000), and Severe Impairment Battery (SIB; Saxton, McGonigle-Gibson, Swihart, Miller, & Boller, 1990; Panisset, Roudier, Saxton, & Boiler, 1994).

Compared to the MMSE, these three tests have shown reduced floor effects in advanced dementia. Although an ability to detect cognitive change over time was established for both the SCIP (Peavy et al., 1996) and the SIB (Black et al., 2007; Feldman et al., 2005; Schmitt et al., 1997; Wild & Kaye, 1998), these tests take 30 minutes or more to administer, and this may be too long for persons with an MMSE-score of 5 or below or for those who are very agitated (Saxton et al., 2005). The SMMSE can be administered in 5 minutes, but has some problems with measuring cognitive change over time (Harrell et al., 2000). It is important that a test for advanced dementia has a short administration time and is able to detect cognitive change over time.

A short version of the SIB (SIB-S) was developed to make the SIB more suitable for persons with a short attention span and thus to minimize floor effects (Saxton et al., 2005). The SIB-S takes approximately 10–15 min to administer, and was validated in several studies (Ahn, Kim, Saxton & Kim, 2007; de Jonghe, Wetzels, Mulders, Zuidema, & Koopmans, 2009; Saxton et al., 2005). Low SIB-S scores were associated with cognitive impairment as measured with the MMSE, dementia severity, and functional dependency in activities of daily living. In addition, very minimal floor effects were evidenced (Ahn et al., 2007; de Jonghe et al., 2009; Saxton et al., 2005). The ability of the SIB-S to measure cognitive change in persons with moderate to severe dementia, however, has not yet been studied.

Our main objective was to examine the ability of the SIB-S to measure cognitive change in institutionalized persons with moderate to severe dementia. To examine to what extent the SIB-S is susceptible to floor and ceiling effects in the long-term, we also took into account the different dementia stages. In addition, as during the early phases of Alzheimer’s disease (AD) the decline of semantic memory is often less pronounced and less consistent compared to episodic memory (Hodges & Patterson, 1995; Perry, Watson, & Hodges, 2000), it is possible that aspects of semantic memory are better preserved in the advanced stages of dementia. Therefore, we explored if the ability of participants to complete semantic, non-semantic, and episodic memory items of the SIB-S was related to their diagnosed dementia stage and to their overall cognitive functioning.

## METHODS

### Subjects

This cohort study was part of a larger prospective cohort study in which participants were enrolled from 14 dementia special care units from 9 nursing homes in the Netherlands (Wetzels, Zuidema, de Jonghe, Verhey, & Koopmans, 2010). This same sample has been used to validate the SIB-S in the Netherlands (de Jonghe et al., 2009). Participants’ elderly care physicians (Koopmans, Pellegrom, & van der Geer, 2017) systematically screened all participants for inclusion. Participants were considered for inclusion provided they: (1) met the Diagnostic and Statistical Manual of Mental Disorders fourth edition criteria for dementia (American Psychiatric Association, 2000); (2) had no life-threatening disease at the time of inclusion; (3) had to reside in a nursing home for at least 4 weeks. Participants were followed for 2 years and all assessments were administered at baseline and subsequently during 4 biannual follow up visits.

### Measures

The course of cognitive functioning was assessed with the Severe Impairment Battery Short version (SIB-S; Saxton et al., 2005). This test, which was developed for the more advanced dementia stages, has a total score ranging from 0 to 50. See Table 1 for a representation of the individual items of the SIB-S per subscale. The SIB-S has been translated into Dutch and has been validated (de Jonghe et al., 2009). Furthermore, the SIB-S has a high reliability and a high concurrent validity with the MMSE (de Jonghe et al., 2009).

**Table 1:** SIB-S items per subscale

Item number	Score
*Social Interaction*	
1. Shake hands	0, 1, 2
2. Follow directions	0, 1, 2
*Memory*	
3. Immediate recall of examiner’s name	0, 1, 2
12. Delayed recall of examiner’s name	0, 1, 2
21. Recall of coloured block	0, 1, 2
25. Delayed recall of cup	0, 1
*Orientation*	
4. Subject’s name	0, 1, 2
7. City name	0, 1, 2
*Language*	
5. Write name	0, 1, 2
6. Copy name	0, 1, 2
8. Responsive naming – cup	0, 1, 2
9. Reading comprehension	0, 1, 2
10. Verbal comprehension	0, 1, 2
13. Confrontational naming – cup	0, 1, 2
15. Object naming – cup	0, 1, 2
17. Forced choice naming – cup	0, 1
18. Confrontational naming – spoon	0, 1, 2
19. Color naming – blue	0, 1, 2
23. Color naming – red	0, 1, 2
*Attention*	
11. Digit span	0, 1, 2
*Praxis*	
14. Using cup – photograph	0, 1, 2
16. Using cup – cup	0, 1, 2
*Visuospatial ability*	
20. Color matching	0, 1, 2
22. Color discrimination	0, 1, 2
*Construction*	
24. Drawing – square	0, 1, 2
*Orienting to name*	
26. Orienting to name	0, 1, 2

To create a domain of items that relied mostly on Semantic memory, we added the Language, Praxis, Visuospatial ability, and Construction subscales, creating a score ranging from 0 to 31. For the Episodic memory domain, we used the Memory subscale minus item 3 (immediate recall of the examiner’s name) with a score ranging from 0 to 5. To create a domain of items that relied least on semantic memory (Memory-Orientation-Attention), we added the Memory, Orientation, and Attention subscales with a score ranging from 0 to 13. In case a participant could not communicate due to severe dementia a total score of 0 was given. If a participant did not cooperate at a particular assessment, this assessment was registered as missing.

Type of dementia diagnosis was established by the elderly care physicians using international consensus criteria (McKhann et al., 1984; Roman et al., 1993) and the Dutch consensus guidelines for AD, vascular dementia (VaD), mixed AD/VaD, and other diagnosis. Independently of the elderly care physicians, one of the authors (R.B.W.) checked eligibility and diagnosis of all participants against their clinical notes. In cases of doubt, consensus meetings were organized to ensure inclusion of participants who met the inclusion criteria. See Table 2 for a representation of dementia subtypes diagnosed in the sample.

**Table 2:** Subtypes of dementia for the whole sample

Subtype	*n*
Possible/probable AD	82
Possible/probable VaD	24
Mixed AD/VaD	11
Mixed AD/not otherwise specified	8
Mixed VaD/not otherwise specified	2
Mixed VaD/Dementia with Lewy Bodies	2
Mixed VaD/Parkinson’s Dementia/not otherwise specified	1
Mixed Dementia with Lewy Bodies/not otherwise specified	1
Mixed Frontotemporal Dementia/not otherwise specified	1
Mixed Parkinson’s Dementia/not otherwise specified	1
Dementia with Lewy Bodies	4
Frontotemporal Dementia	4
Parkinson’s Dementia	1
Not otherwise specified	75

AD, Alzheimer’s disease; VaD, vascular dementia.

The Global Deterioration Scale (GDS) for Primary Degenerative Dementia (Reisberg, Ferris, de Leon, & Crook, 1982) was used to determine the severity/stage of dementia at baseline. This assessment scale consists of a seven-point scale: (1) no cognitive decline; (2) mild memory loss reported by the participant, but the tests show no loss of function; (3) mild cognitive impairment (MCI); (4) early dementia; (5) moderate dementia; (6) moderately severe dementia; and (7) severe cognitive decline/late dementia. In this particular cohort study, only participants with a GDS-score of 5, 6, or 7 were included.

The Mini-Mental State Examination (MMSE; Folstein et al., 1975) was administered for baseline characteristics.

Activities of daily living (ADL) dependency was measured with section G of the Interrai Long-Term Care Facility scale (2005, version 07). For this study, the hierarchical ADL scale was used, which consists of 4 of the ADL items of the Interrai (Morris, Fries, & Morris, 1999). A hierarchical decision tree was used to obtain ADL dependency scores: (0) independent (in all 4 ADL items), (1) supervision, (2) limited, (3) extensive 1, (4) extensive 2, (5) dependent, and (6) total dependence. The hierarchical ADL scale has good to excellent interrater reliability and a Cronbach’s alpha of .90 (Morris et al., 1999).

### Ethical Considerations

This study was approved by the regional research ethics committee and completed in accordance with the Helsinki Declaration. The participants and/or their relatives and legal guardians were informed about the study and gave their written consent.

### Statistical Analysis

The analyses were conducted with Statistical Package for Social Sciences (SPSS) version 23 and R version 3.4.2 (R Core Team, 2017). To compare the baseline characteristics between the different dementia stages (GDS-score), Pearson χ^2^-tests for gender, education, medication use, and ADL dependency were conducted. The data for age, years in nursing home, years in nursing home unit, MMSE-score, and SIB-S score were not normally distributed in all separate GDS-groups. Therefore, non-parametric Independent Samples Kruskal-Wallis Tests were conducted to compare those variables between GDS-groups.

To examine concurrent validity, Kendall’s tau correlations between total SIB-S score and ADL dependency were calculated for all five assessments. To get an indication of how much measured SIB-S scores were spread around a “true” score, we calculated standard error of measurement (SEM) for all five assessments with the following formula: SEM = S* √(1–r_kk_).

Growth curve models were used to measure the course of SIB-S scores over time. These models can handle missing data and take into account multiple assessments within one subject. The standard model consisted of two levels: SIB-S scores at level 1, nested within subjects (level 2). Time (measured in days between T0 (representing 0 days) and T1, T0 and T2, T0 and T3, and T0 and T4) was the continuous independent variable and age at baseline (centered) was added as additional covariate.

To assess whether the course of SIB-S scores over time showed non-linearity, an additional quadratic Time regressor was added to the standard model. To analyze the difference in the course of SIB-S scores over time between dementia stages, GDS-score (3 categories) and a GDS-score by Time interaction were added as independent variables to the standard model. In case of a significant GDS-score by Time interaction, separate models for GDS-score were run to analyze the effect of Time on SIB-S scores.

To analyze if there were different effects for SIB-S domains, above mentioned growth curve models were also run for the domains Semantic memory, Episodic memory, and Memory-Orientation-Attention.

All models included a random intercept across participants. Random slopes for Time (heterogeneous first-order autoregressive structure) were added if the Likelihood Ratio Test between the random intercept model and the model with additional random slopes was significant (*p* < .05).

A Pearson χ^2^-test was conducted to examine if the number of dropouts at the fifth assessment differed between GDS-groups. In addition, a one-way analysis of variance (ANOVA) was conducted to assess for significant differences in the percentage of total missing assessments between different GDS-groups. Furthermore, a one-way ANOVA, Pearson χ^2^-test or Mann-Whitney *U* (dependent on the outcome variable) was conducted to check whether there were differences in baseline characteristics between participants that completed and participants that did not complete the fifth assessment.

To check if GDS-score at baseline could predict the amount of individual variability in SIB-S scores over time, we conducted a non-parametric Independent Samples Kruskal-Wallis Test. To characterize individual progression characteristics of SIB-S scores over time, we performed a linear regression analysis [with SIB-S as dependent and Time (in days) as predictor] for each separate participant with at least three SIB-S assessments. The residual sums-of-squares (rss) was taken as the variability of the decline. To correct for missing assessments, we divided the rss by the number of assessments. In case of a significant effect of GDS-score on the variability of decline, Independent Samples Mann-Whitney *U* Tests were conducted to check which GDS-groups differed in the variability of decline.

To gain insight about the relationship between individual items of the SIB-S and overall cognitive functioning measured with the SIB-S, items were first ranked by how many participants could still successfully complete the item. As a measure for the items’ completion rate, per item the mean normalized SIB-S score was calculated, where the average and associated standard deviation were taken over all participants and all tests. Normalization was necessary before ranking, because item 17 and 25 are on a 0–1 scale, whereas the rest of the items are on a 0–1–2 scale. After ranking the items, test subjects were separated into 6 groups by their total SIB-S score. The scores 0 and 50 were each taken as a separate group, because these scores would show no variation in individual item scores. The rest of the population was separated in 4 equally sized groups. Then we plotted per item the completion rate by different stages of overall cognitive functioning. The plots show the items ranked based on the whole population, but with the mean normalized SIB-S score per item per group.

Alpha-level was set at .05. For *post hoc* analyses, Bonferroni correction was applied (*α* = .017).

## RESULTS

### Sample Characteristics

A total of 290 participants were eligible for inclusion. For 15 participants, no SIB-S data were available, and 29 participants did not meet inclusion criteria for this particular cohort. Twenty-nine participants scored zero on all SIB-S assessments. These participants probably did not understand test instructions at any assessment, and were excluded from further analyses. In the 217 remaining participants, no significant gender, age, education, or medication use differences were found between GDS-groups at baseline. As expected, MMSE-scores and SIB-S scores differed between GDS-groups, where persons with severe dementia had the lowest mean scores on the MMSE and SIB-S. Furthermore, persons with more severe dementia were on average most ADL dependent and were institutionalized the longest. See Table 3 for baseline characteristics and comparison between GDS-groups.

**Table 3:** Baseline characteristics of the sample

Baseline characteristics	Total sample (*N* = 217)	GDS-score 5 (*n* = 59)	GDS-score 6 (*n* = 108)	GDS-score 7 (*n* = 50)	*p*-value	*η^2^*/*V*
Age in years						
Mean (*SD)*	83.91 (6.82)	83.89 (6.44)	83.35 (6.43)	85.13 (7.97)	.274	*η* ^*2*^=.003
Median	84.47	84.76	83.78	85.82		
Range	59.30–102.20	59.30–97.62	60.51–96.58	65.51–102.20		
Gender, men (%)	23.5	30.5	23.1	16	.216	*V*=.121
Education (%)					.284	*V*=.200
Primary	26.3	20.3	24.1	38		
Low vocational secondary	19.4	20.3	21.3	14		
Mid vocational secondary	6.9	6.8	8.3	4		
High vocational secondary	2.3	5.1	1.9	0		
University	.9	0	.9	2		
Missing values	44.2	47.5	43.5	42		
Years in nursing home	*n*=213	*n*=57	*n*=106	*n*=50	<.001	*η^2^*=.072
Mean (*SD*)	2.69 (2.01)	2.15 (2.00)	2.57 (1.96)	3.55 (2.05)		
Median	2.06	1.59	1.96	3.61		
Range	.10–10.28	.12–10.28	.18–9.08	.10–8.27		
Years in nursing home unit	*n*=213	*n*=57	*n*=106	*n*=50	<.001	*η^2^*=.095
Mean (*SD*)	2.36 (1.94)	1.80 (1.84)	2.22 (1.84)	3.32 (1.95)		
Median	1.81	1.16	1.71	2.78		
Range	.05–10.28	.05–10.28	.17–9.08	.10–7.57		
Medication use (%)						
Antipsychotic	35.0	32.2	32.4	44.0	.332	*V*=.103
Antidepressant	27.6	22	31.5	26.0	.412	*V*=.091
Hypnotic	13.8	13.6	14.8	12.0	.935	*V*=.033
Anxiolytic	18.4	15.3	17.6	24.0	.492	*V*=.083
NMDA inhibitor	5.5	8.5	4.6	4.0	.586	*V*=.079
ADL dependency						
Independent	2.3	5.1	1.9	0	<.001	*V*=.390
Supervision	12	28.8	8.3	0		
Limited	6.9	8.5	9.3	0		
Extensive 1	33.6	35.6	38.9	20.0		
Extensive 2	6.0	5.1	5.6	8.0		
Dependent	27.2	16.9	27.8	38.0		
Total dependence	12.0	0	8.3	34.0		
MMSE	*n*=213	*n*=59	*n*=105	*n*=49	<.001	*η^2^*=.528
Mean (*SD*)	7.19 (6.06)	13.08 (4.79)	6.75 (4.74)	1.02 (1.93)		
Median	7	13	7	0		
Range	0-24	1-24	0–17	0–7		
SIB-S						
Mean (*SD*)	27.50 (15.48)	40.44 (7.04)	29.49 (12.12)	7.94 (8.74)	<.001	*η^2^*=.528
median	31	42	32	4.5		
range	0–50	16–50	0–48	0–32		

*Note.* GDS-score 5, moderate dementia; GDS-score 6, moderately severe dementia; GDS-score 7, severe cognitive decline/late dementia; *p*-value, results of the Pearson *χ²*-test for gender, education, medication use, ADL dependency, and Independent Samples Kruskal-Wallis Test for age, years in nursing home (unit), MMSE-score, and SIB-S score to compare between GDS-scores; *η^2^*, eta squared, effect size for Independent Samples Kruskal-Wallis Test; *V*, Cramer’s V, effect size for Pearson ‘s *χ²*-test. N/n, number of participants; SD, standard deviation; MMSE, Mini-Mental State Examination; SIB-S, Severe Impairment Battery Short version.

A minimum of 1 SIB-S assessment (*n* = 217) and a maximum of 5 SIB-S assessments (*n* = 55) were available for each participant. Twenty participants had missing data at an assessment before their last assessment; thus, the number of participants at T4 (*n* = 70) was different from the number of participants that completed all 5 SIB-S assessments. Participants missed an average of two of five assessments. Reasons for dropping out were the move to another institution, death, too ill to be tested, or refusal of the participant to further cooperate. Participants who dropped out were older (on average 2.73 years; *F*(1,216) = 7.834; *p* = .006; *η* ^*2*^ = .035) and were more likely to use hypnotics (χ^2^(1) = 11.889; *p* = .020; *η* ^*2*^ = .026) than participants who completed the fifth assessment. There were no other significant differences in baseline characteristics between participants who completed and participants who dropped out at the fifth assessment.

### Concurrent Validity

Low scores on SIB-S were associated with high ADL dependency scores on all five assessments, (T0: Kendall’s tau = -.363, *p* < .001; T1: Kendall’s tau = -.452, *p* < .001; T2: Kendall’s tau = -.478, *p* < .001; T3: Kendall’s tau = -.558, *p* < .001; T4: Kendall’s tau = -.665, *p* < .001).

### SEM

For the SIB-S, the SEM was 3.21 points for T0, 2.89 points for T1, 2.91 points for T2, 2.96 points for T3, and 2.62 points for T4.

### Growth Curve Models

For coefficients and standard deviations for all growth curve models, see Table 4.

**Table 4:** Growth curve models for SIB-S total and domain scores

	Total SIB-S	Semantic memory	Episodic memory	M-O-ATT
**Effect of Time for the whole sample**				
Fixed effects (coefficients and *SE*)				
Constant	27.902(1.073)	17.122(.734)	1.555(.096)	6.199(.264)
Age (centered)	.041 (.155)	.030(.105)	−.018(.013)	.008(.038)
Time (days from T0)	−.012(.001)^***^	−.008(.001)^***^	−.0008(.0002)^***^	−.002(.0003)^***^
**Effect of Time² for the whole sample**				
Fixed effects (coefficients and *SE*)				
Constant	27.748(1.082)	17.060(.740)	1.499(.099)	6.157(.268)
Age (centered)	.042(.155)	.030(.105)	−.018(.013)	.008(.038)
Time (days from T0)	− .009(.003)^**^	−.006(.002)^**^	.0002(.0005)	−.002(.001)
Time²	<−.0001(<.0001)	< .0001(<.0001)	< .0001(<.0001)^*^	< .0001(<.0001)
**Effect of GDS x Time**				
Fixed effects (coefficients and *SE*)				
Constant^a^	29.896(.999)	18.369(.713)	1.558(.110)	6.523(.257)
Age (centered)	.171(.102)	.113(.072)	−.010(.010)	.042(.026)
Time (days from T0)^a^	−.018(.002)^***^	−.012(.001)^***^	−.001(.0002)^***^	−.004(.0006)^***^
GDS (6 compared to 5)	11.434(1.673)^***^	7.705(1.194)^***^	1.126(.184)^***^	3.062(.431)^***^
GDS (6 compared to 7)	−21.970(1.774)^***^	−14.380(1.267)^***^	−1.334(.196)^***^	−4.903(.457)^***^
GDS (7 compared to 5)	33.404(1.984)^***^	22.086(1.417)^***^	2.460(.218)^***^	7.966(.512)^***^
GDS (6 compared to 5) × Time	.009(.003)^**^	.006(.002)^**^	.0002(.0004)	.001(.0009)
GDS (6 compared to 7) × Time	.013(.003)^***^	.009(.002)^***^	.0009(.0004)^*^	.003(.0009)^**^
GDS (7 compared to 5) × Time	−.004(.004)	−.003(.003)	−.0007(.0004)	−.002(.001)
**Effect of Time for GDS-score 5**				
Fixed effects (coefficients and *SE*)				
Constant	41.330(.910)	26.033(.693)	2.682(.152)	9.591(.259)
Age (centered)	−.127(.134)	−.079(.097)	−.048(.021)^*^	−.042(.038)
Time (days from T0)	−.008(.003)^**^	−.004(.002)	−.0008(.0003)^**^	−.002(.0007)^**^
**Effect of Time for GDS-score 6**				
Fixed effects (coefficients and *SE*)				
Constant	30.046(1.177)	18.472(.848)	1.570(.127)	6.560(.297)
Age (centered)	.421(.181)^*^	.293(.130)^*^	.011(.018)	.103(.045)^*^
Time (days from T0)	−.018(.002)^***^	−.012(.002)^***^	−.001(.0003)^***^	−.004(.0007)^***^
**Effect of Time for GDS-score 7**				
Fixed effects (coefficients and *SE*)				
Constant	8.045(1.271)	4.071(.789)	.210(.066)	1.643(.324)
Age (centered)	.031(.154)	.011(.095)	−.008(.007)	.012(.039)
Time (days from T0)	−.004(.001)^**^	−.002(.001)^*^	−.0002(.0002)	−.0007(.0005)

*Note.* GDS-score 5, moderate dementia; GDS-score 6, moderately severe dementia; GDS-score 7, severe cognitive decline/late dementia

a Group with GDS-score 6 was used as reference group.

* *p* < .05.

** *p* < . 01.

*** *p* < .001.

SIB-S, Severe Impairment Battery Short version; M-O-ATT, Memory-Orientation-Attention domain.

#### Course of total SIB-S scores over time

Average total SIB-S scores declined significantly over time, *F*(1,114.058) = 65.240, *p* < .001. The addition of a quadratic time variable (Time²) to this model was not significant, χ^2^(1) = 1.245, *p* > .05. See Table 5 for number of participants and mean SIB-S scores per assessment. Not every participant declined in SIB-S scores between two assessments. Of the 217 participants, 152 had more than one assessment; whereas 67.1% of the participants scored lower, 9.2% were equivalent, and 23.7% scored higher at their last assessment in comparison to their first assessment.

**Table 5:** Mean SIB-S scores on successive assessments for the whole sample

	*N*	Mean (SD)	Range
T0	217	27.50 (15.48)	0–50
T1	149	26.81 (17.61)	0–50
T2	117	23.49 (18.03)	0–50
T3	92	20.97 (17.66)	0–49
T4	70	19.93 (18.99)	0–50

*N*, number of participants; SD, standard deviation.

#### Effect of baseline dementia stage (GDS-score) on the course of SIB-S total scores over time

**Fig. 1:**
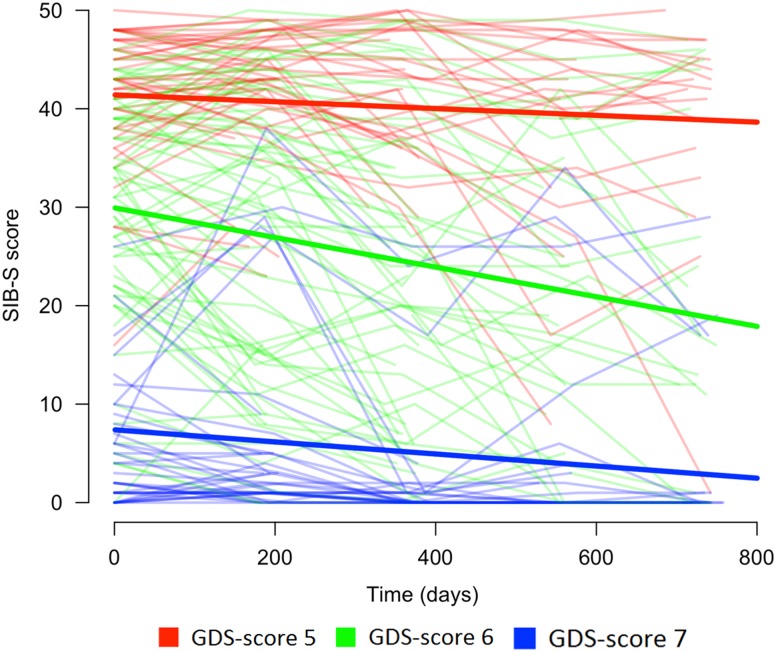
Course of SIB-S scores over time for persons with moderate to severe dementia according to dementia stage (without a correction for age). The bold lines represent the mean course of SIB-S scores per GDS-score and the thin lines represent the course of SIB-S scores for each individual participant.

Baseline GDS-score had an effect on the rate of decline in SIB-S scores over time, *F*(2,104.960) = 8.517, *p* < .001. Mean SIB-S scores of persons with GDS-score 6 declined significantly faster than persons with GDS-score 5 (*t*(110.993) = 2.700; *p* = .008) or GDS-score 7 (*t*(103.490) = 3.912; *p* < .001). By contrast, there was no significant difference in the course of SIB-S scores over time between persons with GDS-score 5 and persons with GDS-score 7 (*t*(99.799) = 1.083; *p* = .282). The average decline on the SIB-S per half year (182.5 days) was 1.41 points for persons with GDS-score 5 (*F*(1,37.789) = 8.597; *p* = .006), 3.26 points for persons with GDS-score 6 (*F*(1,49.912) = 51.533; *p* < .001), and .82 points for persons with GDS-score 7 *(F*(1,119.121) = 9.559; *p* = .002). See Figure 1 for a visual representation of the progression of SIB-S scores over time for each dementia stage. As can be seen in Figure 1, there is considerable variability between individual participants with the same GDS-score in their course of SIB-S scores over time.

In addition, an Independent Samples Kruskal-Wallis Test showed a significant difference between GDS-groups in individual variability in SIB-S scores over time, *H* = 20.332, *p* < .001, *η* ^*2*^ = .150. Persons with GDS-score 7 had less individual variability than persons with GDS-score 5 (*U* = 313.000; *p* = .004; *η* ^*2*^ = .035) or GDS-score 6 (*U* = 427.000; *p* < .001; *η* ^*2*^ *= .*208).

The number of dropouts at the fifth assessment was significantly different between persons with GDS-score 5 (64.4%), GDS-score 6 (75%), and GDS-score 7 (56%), Pearson χ^2^(2) = 5.520, *p* = .049, *V* = 0.159. However, there was no significant difference in the percentage of total missing assessments between persons with GDS-score 5 (39.3%), GDS-score 6 (44.3%), and GDS-score 7 (34%), *F*(2,214) = 1.862, *p* = .158, *η* ^*2*^ *= .*017.

#### Course of SIB-S domains over time

For the whole sample, there was a significant average decline in Semantic memory (*F*(1,115.267) = 55.425; *p* < .001), Episodic memory (*F*(1,476.310) = 26.045; *p* < .001), and Memory-Orientation-Attention (*F*(1,451.032) = 61.151; *p* < .001).

Baseline GDS-score had a significant effect on the rate of decline in Semantic memory (*F*(2,106.473) = 9.418; *p* < .001) and Memory-Orientation-Attention (*F*(2,92.064) = 4.992; *p* = .009), but a non-significant effect on Episodic memory (*F*(2,496.335) = 3.103; *p* = .050). As the effect on Episodic memory was marginally significant, we also conducted *post hoc* tests for this subscale. In accordance with total SIB-S scores, Semantic memory declined significantly faster for persons with GDS-score 6 compared to persons with GDS-score 5 (*t*(109.734) = 2.856; *p* = .005) or GDS-score 7 (*t*(107.059) = 4.102; *p* < .001). For Episodic memory, however, only persons with GDS-score 6 declined significantly faster than persons with GDS-score 7, *t*(492.516) = 2.437, *p* = .015. In addition, also for Memory-Orientation-Attention only persons with GDS-score 6 declined significantly faster than persons with GDS-score 7, *t*(93.067) = 3.197, *p* = .002. For decline of domains per GDS-score, see Table 4.

### Relation Between Individual Items and Overall Cognition

**Fig. 2:**
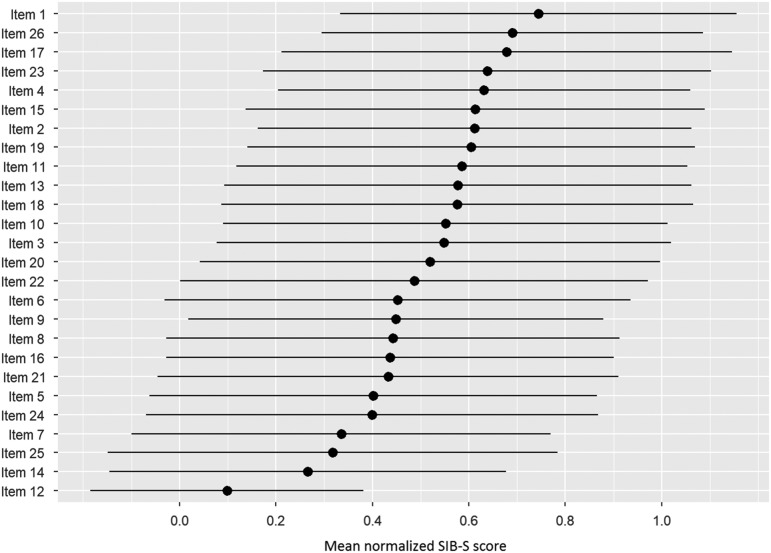
Ranking of the items by how many participants could still successfully complete the item. As a measure for the items completion rate, per item the mean normalized SIB-S score was calculated. The average (represented by bullets) and associated standard deviation (represented by lines) were taken over all participants and all tests.

Figure 2 shows the ranking of the normalized mean score of the SIB-S items by how many patients could still successfully complete the item. The response on item 1 (shake hands) was mostly correct, while the response on item 12 (delayed recall of examiner’s name) was mostly incorrect. The large standard deviation of the mean normalized score of the items indicates substantial variation between participants in their response on the items. Furthermore, it was mainly the items concerning episodic memory (item 12 and 25) and praxis/construction (items 5, 14, and 24) that were the hardest for participants with moderate to severe dementia.

**Fig. 3:**
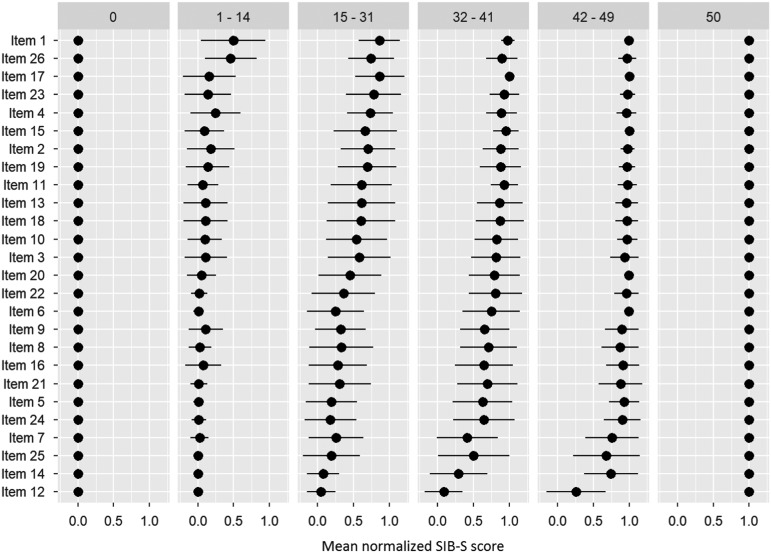
Ranking of the items by how many participants could still successfully complete the item per group (classified by total SIB-S score). The ranking was based on the whole population. As a measure for the item’s completion rate, per item the mean normalized SIB-S score was calculated per group. The mean normalized SIB-S score was calculated by taking the average (represented by bullets) and associated standard deviation (represented by lines) over all participants within one group.

After ranking the items, we separated our participants into six groups based on their total SIB-S score: 0 (*n* = 78), 1–14 (*n* = 138), 15–31 (*n* = 140), 32–41 (*n* = 135), 42–49 (*n* = 148), and 50 (*n* = 6). Figure 3 shows the items ranked based on the whole population, but with the mean normalized SIB-S score per item per group. The pattern found earlier for the whole sample can also be seen in the ranking by subgroups. The variation of the mean normalized score of the items was smaller when divided by subgroups, especially in the groups with the lowest and highest total SIB-S scores. The hardest items showed little or no variation in the group with a SIB-S score 1–14. For example, all the participants with a SIB-S score 1–14 had an incorrect response on items concerning episodic memory (items 12 and 25) and praxis/construction (items 5, 6, and 14). In the group with a SIB-S score 42–49, the variation was the smallest for the easiest items.

## DISCUSSION

This is the first study that examined the ability of the SIB-S to measure cognitive change in institutionalized persons with moderate to severe dementia. Mean total scores on the SIB-S declined over a 2-year period, and the rate of decline differed between dementia stages at baseline. The decline of SIB-S scores was more rapid for persons with moderately severe dementia than for persons with moderate or severe dementia suggesting a modest floor and slight ceiling effect for the SIB-S. Furthermore, we explored if the ability of participants to complete semantic, non-semantic, and episodic items of the SIB-S was related to their diagnosed dementia stage and to their overall cognitive functioning. For semantic memory items, the pattern of decline for different dementia stages was similar as for SIB-S total scores. However, for episodic and non-semantic memory items, only persons with moderately severe dementia declined faster than persons with severe dementia.

While we observed a decline for all three item categories for persons with moderately severe dementia, we only observed a decline in episodic and non-semantic memory items in persons with moderate dementia and a decline in semantic memory items in persons with severe dementia. In addition, our item rankings showed that persons with the lowest overall cognitive functioning had severe problems with items regarding episodic memory and praxis.

The decline over time found in persons with moderate to severe dementia suggests that mapping the course of cognitive functioning with the SIB-S is possible in this population. The faster decline in SIB-S scores for persons with moderately severe dementia than for person with moderate or severe dementia indicates, however, a modest floor and a slight ceiling effect. For some participants with moderate dementia, the questions of the SIB-S were probably too easy to adequately map their cognitive abilities. Indeed, Figure 1 shows that a larger proportion of the participants in the group with moderate dementia had relatively higher, more stable SIB-S scores than in the group with moderately severe dementia. This was probably due to the high number of semantic items in the SIB-S.

Studies have shown that mild AD patients were able to visually recognize and correctly name and match words and pictures (word-picture matching), a finding that suggests that aspects of semantic memory were still intact (Hirono et al., 2001; Hodges & Patterson, 1995). Probably, aspects of semantic memory are also intact in the moderate stage of dementia, and these aspects deteriorate mainly in the moderately severe stage of dementia. It was indeed only semantic memory that showed an accelerated decline in SIB-S scores in the moderately severe dementia group in comparison to the moderate dementia group.

Additionally, for some participants with severe dementia, the questions of the SIB-S were probably too difficult. For persons with a baseline SIB-S score close to zero, it is not possible to decline much further in SIB-S scores. As can be seen in Figure 1, part of the participants in the group with severe dementia scored close to zero at every assessment. Furthermore, in persons with severe dementia, we only observed a decline in semantic memory. Our item ranking showed that two episodic memory items were impossible for persons with the lowest overall cognitive scores. It is possible that semantic memory is the only memory system that can decline when persons are in the severe stage of dementia. Therefore, the small non-semantic part of the SIB-S could also have contributed to the slower decline in SIB-S scores in the severe dementia group in comparison to the moderately severe dementia group. Additional studies with tests specifically developed to tap into different types of memory in advanced dementia may provide additional insight.

A similar pattern, a slower decline in cognitive functioning in the groups with the lowest and highest baseline-scores in comparison to the middle group, has also been found for other cognitive tests for dementia, including the original SIB (Schmitt et al., 1997), MMSE (Morris et al., 1993), and Alzheimer’s Disease Assessment Scale (Stern et al., 1994). Only the population included in the study of the original SIB was comparable to our population, while the other studies also contained participants with mild dementia and had fewer participants with very severe dementia. Therefore, it seems difficult to draw conclusions about what dementia stage shows the fastest cognitive decline.

It seems more likely that found differences in rate of cognitive decline between dementia stages are a test effect instead of a population effect. An alternative explanation is that in our study the group with moderately severe dementia at baseline had the largest score range on the SIB-S. The value of a correlation between variables can be larger when there is more variability among test scores (Goodwin & Leech, 2006). Although the SIB-S thus seems most suitable in the moderately severe stage of dementia, our finding of decline in all examined dementia stages suggests that the SIB-S is a useful tool to track cognition in the entire range of moderate to severe dementia.

There was, however, considerable individual variability in total SIB-S scores over time, especially among participants with moderately severe dementia at baseline. A striking finding was the relatively high percentage (23.7%) of participants that had a SIB-S score that was higher at their last than at their first assessment. One of the explanations for the variability in SIB-S scores is that cognitive fluctuations (spontaneous alterations in cognition, attention, and arousal) are quite common among persons with dementia (Lee, Taylor, & Thomas, 2012).

Second, we did not control for time of day participants were tested. If a participant was most lucid in the morning, but was tested at different times of the day across assessments, this could have affected the results. Furthermore, participants received treatment when necessary. This was, however, not registered nor controlled for. If a significant number of participants received treatment with a positive effect on cognitive functioning, this could have led to the relatively high percentage of participants that improved on the SIB-S during our study. Future studies into the course of cognitive functioning in dementia should control for time of day of assessments, and whenever possible, also for received treatments with an effect on cognitive functioning.

Next to cognitive fluctuations, the individual variability in total SIB-S scores could also be caused by the relatively high SEM of the SIB-S. For example, for the first assessment, the 68% confidence interval around a participant’s “true” SIB-S score was ±3.21 points. Thus individual variability on the SIB-S could partly be explained by fluctuations around a participant’s “true” score. One method to estimate the probability that an individual’s change in test scores is not due to chance is calculating the Reliable Change Index (RCI) for that particular test (Chelune, Naugle, Lüders, Sedlak & Awad, 1993). Unfortunately, to calculate the RCI the test–retest reliability coefficient is required, which was not available in our study. Future studies could calculate the RCI for the SIB-S to draw stronger conclusions about what represents clinically significant decline in dementia.

There were some other limitations. There were far more women than men in our study. Women tend to have a faster age-related cognitive decline and a higher risk of developing AD than men (Li & Singh, 2014). The imbalance in gender could have affected results. However, women live longer than men on average, and consequently there are more women than men with advanced dementia in institutions. Therefore, our sample is representative for institutionalized persons with dementia, but caution is warranted when generalizing our results to institutionalized men with dementia.

Another limitation was that only 70 of the 217 participants completed the last assessment. Main reasons for dropping out were death and terminal disease. Participants that dropped out were on average older than participants that completed the last assessment. Unfortunately, this was a consequence of the included population, persons with more advanced dementia. To still include all participants in our analyses, we analyzed our data with mixed models to handle missing data.

Dementia subtype was not specifically examined in this study. A specific dementia diagnosis was often inconclusive as neuroimaging data were not routinely available. Neuroimaging can help to distinguish different dementia subtypes more reliably (Health Quality Ontario, 2014). However, it is not common practice to use neuroimaging for the diagnosis of dementia subtypes in institutionalized persons. Furthermore, distinguishing between dementia subtypes becomes harder above the age of 80 as there is mainly mixed pathology in this group (Fotuhi, Hachinski, & Whitehouse, 2009). So, we believe our results bear relevance to institutionalized populations with moderate to severe dementia. The course of cognitive functioning can be affected by dementia subtype; however, the effects of dementia subtype on the course of SIB-S scores warrant further research.

In conclusion, this is the first study that used the SIB-S to examine the course of cognitive functioning across different dementia stages. We found that it is feasible to measure cognitive decline in advanced dementia. Although certain SIB-S items regarding praxis and episodic memory will provide no additional information in persons with the lowest cognitive functioning, we recommend administering all SIB-S items in every dementia stage. It is a relatively short test to administer and there is a preconceived order in the SIB-S items. For persons in the moderate dementia stage who score near maximum on the SIB-S, additional tests less focused on semantic memory could be necessary to get a complete picture of their cognitive functioning at that moment. However, we recommend using the SIB-S from the moment a person reaches the moderate stage of dementia as the SIB-S is one of few available tests that can still track cognitive functioning even when a person eventually reaches the severe stage of dementia.
